# Unique Tandem Repeats in the Inverted Terminal Repeat Regions of Monkeypox Viruses

**DOI:** 10.1128/spectrum.03199-22

**Published:** 2023-03-28

**Authors:** Perumal Arumugam Desingu, K. Nagarajan, Nagalingam R. Sundaresan

**Affiliations:** a Department of Microbiology and Cell Biology, Indian Institute of Science, Bengaluru, India; b Department of Veterinary Pathology, Madras Veterinary College, Tamil Nadu Veterinary and Animal Sciences University (TANUVAS), Vepery, Chennai, Tamil Nadu, India; University of Prince Edward Island

**Keywords:** monkeypox, multicountry outbreak 2022, adaptive evolution, tandem repeats, transposon, horizontal gene transfer

## Abstract

The genetic diversity, especially in noncoding regions between clade I, clade IIa, and clade IIb monkeypox viruses (MPXVs), is still not fully understood. Here, we report that unique 16-nucleotide-length tandem repeats in MPXVs viruses are located in the noncoding regions of inverted terminal repeats (ITR), and the copy number of this repeat is different among clade I, clade IIa, and clade IIb viruses. It is noteworthy that tandem repeats containing these specific sequences (AACTAACTTATGACTT) are only present in MPXVs and are not found in other poxviruses. Also, the tandem repeats containing these specific sequences (AACTAACTTATGACTT) do not correspond to the tandem repeats present in the human and rodent (mice and rat) genomes. On the other hand, some of the reported tandem repeats in the human and rodent (mice and rat) genomes are present in the clade IIb-B.1 lineage of MPXV. In addition, it is noteworthy that the genes flanking these tandem repeats are lost and gained compared between clade I, clade IIa, and clade IIb MPXV.

**IMPORTANCE** The different groups of MPXVs contain unique tandem repeats with different copy numbers in the ITR regions, and these repeats may be likely to play a role in the genetic diversity of the virus. Clade IIb (B) MPXV contains 38 and 32 repeats similar to the Tandem repeats reported in the human and rodent genome, respectively. However, none of these 38 (human) and 32 (rodent) tandem repeats matched the tandem repeats (AACTAACTTATGACTT) found in the present study. Finally, when developing attenuated or modified MPXV vaccine strains, these repeats in noncoding genomic regions can be exploited to incorporate foreign proteins (adjuvants/other virus proteins/racking fluorescent proteins such as green fluorescent protein) to carry out studies such as vaccine production and virus pathogenesis.

## OBSERVATION

**FIG 1:**
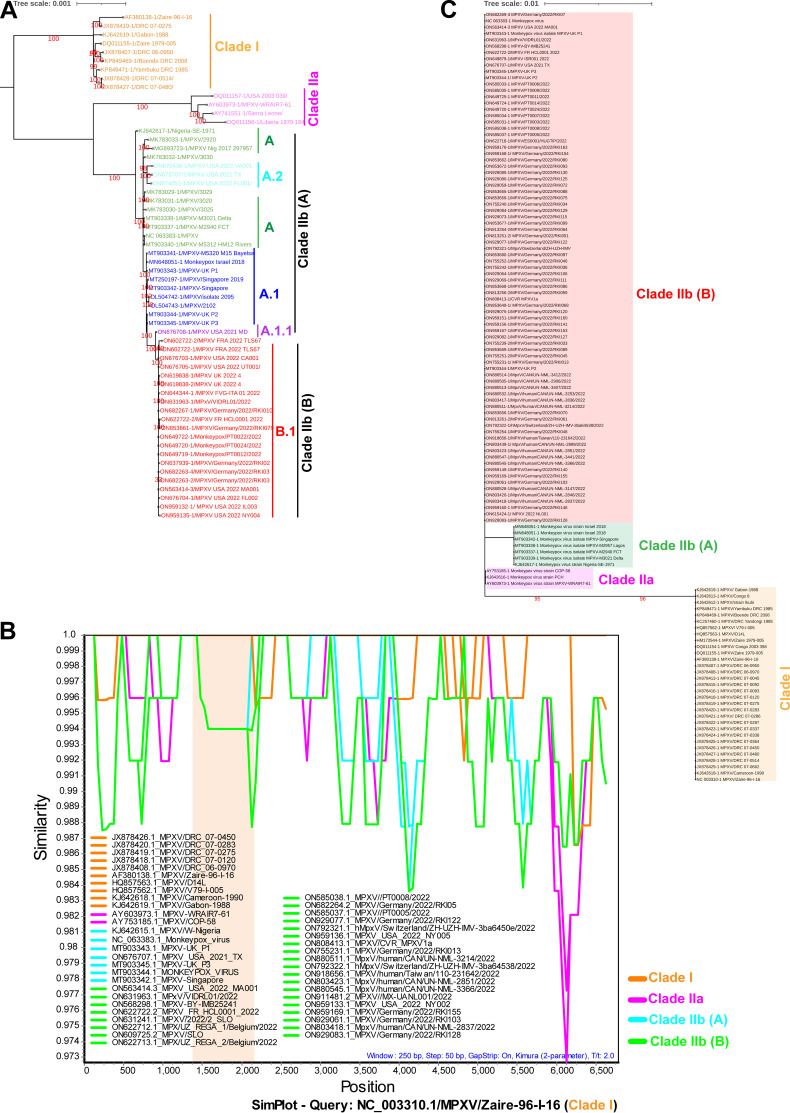
Genetic diversity in the monkeypox viruses. (A) In the complete genome-base phylogenetic analysis, similar to the previous core SNP alignment-based (1) analysis and ITR based analysis (2), MPXVs-2022 viruses formed a distinct group B.1. Similarly, other A, A.1. A.1.1, and A.2 groups were also clustered in the complete genome-base phylogenetic analysis, similar to the previous core SNP alignments-based analysis. (B) Similarity plots display the ≈600-bp-long region of nucleotide sequence diversity (gap) in the 3′-ITR regions between clade I, clade IIa, clade II (A), and clade II (B) MPXVs. NC_003310.1/MPXV/Zaire-96-I-16 (clade I) was used as the query reference sequence. (C) Phylogenetic analysis for 3′-ITR regions containing tandem repeats. clade I, clade IIa, clade IIb (A), and clade IIb (B) MPXVs formed a separate group. (Details of the sequences used for this analysis are presented in Data set S1 and S2.)

**FIG 2:**
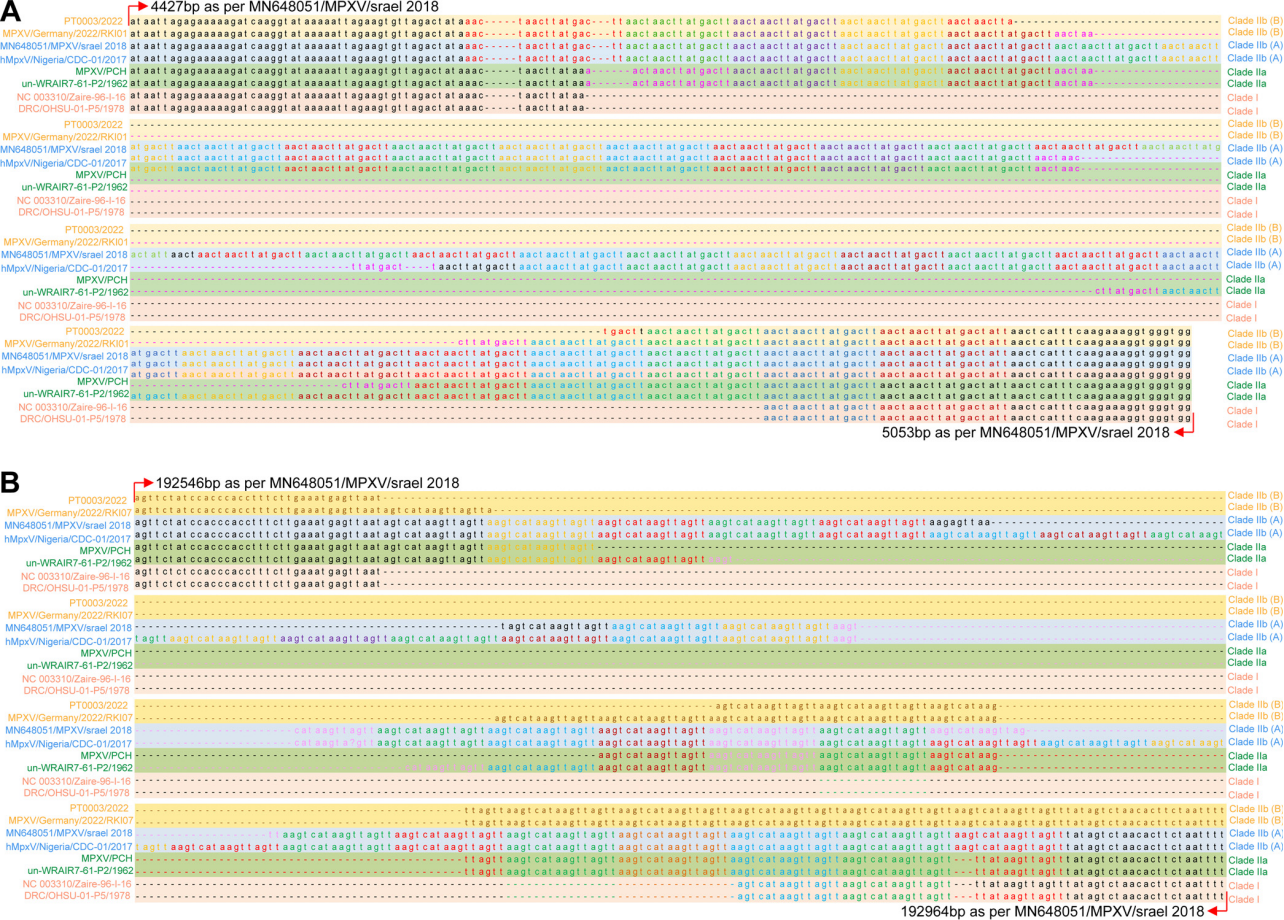
Displaying the unique tandem repeats in the MPXVs. (A and B) Comparison of copy numbers of tandem repeats in 5′-ITR (A) and 3′-ITR (B) regions of different MPXV viruses. (Details of the sequences used for this analysis are presented in Data set S1 and S2.)

A recent study classified CA-monkeypox viruses (MPXVs) into clade 1/clade I, WA-MPXVs into clade 2/clade IIa, MPXVs-2022 viruses, and closely related viruses into clade 3/clade IIb using core single nucleotide polymorphism (SNP)-based (1) and inverted terminal repeats (ITR)-based (2) analysis. Similarly, our previous study reported that CA-MPXVs/clade I, WA-MPXVs/clade IIa, clade IIb (A) (detected before 2022), and MPXVs-2022/clade IIb (B) viruses fall into four distinct clades based on complete genome sequence-level phylogenetic analysis (3). This study's complete genome-scale phylogenetic analysis of clade 3/clade IIb viruses divided them into A, A.1. A.1.1, B.1, and A.2 groups, similar to core SNP alignment-based (1) and ITR-based (2) analysis; of these, MPXVs-2022 viruses formed a distinct B.1/clade IIb (B) group (Fig. 1A). Subsequently, when the 5′-ITR and 3′-ITR regions in MPXVs were subjected to phylogenetic analysis, we observed that clade I, clade IIa, clade IIb (A), and clade II (B) viruses separated into four distinct groups, similar to the complete genome-scale phylogenetic analysis (Fig. S1A and B). From these, it appears that there is significant genetic diversity in some parts of the ITR between clade I, clade IIa, clade IIb (A), and clade II (B) viruses, so we are interested to find out where this genetic diversity is. For this, we subjected the 5′-ITR and 3′-ITR regions in MPXVs to the similarity plot analysis. This analysis revealed a nearly 600-bp-long region of nucleotide sequence diversity (gap) between clade I, clade IIa, clade IIb (A), and clade II (B) viruses in the 5′-ITR (Fig. S1A in the supplemental material) and 3′-ITR (Fig. 1B) regions. After this, we subjected the sequences of this ITR region, which has genetic diversity, to phylogenetic analysis. In this analysis, clade I, clade IIa, clade IIb (A), and clade IIb (B) viruses formed a separate group at this region of 5′ ITR (Fig. S2B) and 3′ ITR (Fig. 1C). Furthermore, when the genetically diversified 5′-ITR and 3′-ITR regions were analyzed in depth, it was revealed that tandem repeats were present in these regions (Fig. 2A and B; Data set S1 and S2). Subsequent analysis revealed two copies of tandem repeats in clade I viruses, 5 to 12 copies in clade IIa viruses, 28 to 32 copies in clade IIb (A) viruses, and 7 copies in clade IIb (B) viruses (Fig. 2A and B; Data set S1 and 2). We then performed NCBI BLAST analysis to determine whether these tandem repeats (AACTAACTTATGACTT) exist in other poxviruses. This analysis revealed that such tandem repeats with these specific sequences (AACTAACTTATGACTT) are absent in other poxviruses.

Next, we were interested in finding out which of the tandem repeats in the human and rodent (mice and rat) genomes are present in MPXV-2022 viruses. This analysis revealed that clade IIb (B) MPXV contains 38 and 32 repeats similar to the tandem repeats reported in the human and rodent genome, respectively (Table S1 and 2). However, none of these 38 (human) and 32 (rodent) tandem repeats matched the tandem repeats (AACTAACTTATGACTT) found in the present study (Table S1 and S2). At present, the function of these unique tandem repeats (AACTAACTTATGACTT) in MPXV viruses is unknown, and large-scale studies are needed in the future to discover and speculate that these may help predict and prepare for future virus evolution and outbreaks.

Remarkably, in MPXVs-2022 viruses, these tandem repeats are located at 4,678 to 4,789 bp (as per ON676705.1/MPXV_USA_2022_UT001) in the 5′-ITR region (AACTAACTTATGACTT) and 192,376 to 192,487 bp (as per ON676705.1/MPXV_USA_2022_UT001) in the 3′-ITR region (AAGTCATAAGTTAGTT). In addition, it is worth noting that this tandem repeat nucleotide position is in the noncoding intergenic region between MPXVgp003 [Ankyrin (Cop-C19L) J3L] and MPXVgp004 [Ankyrin (CPXV-017) D1L] protein in ITR. In particular, in our previous study, we reported loss and gain of genes at the end of the 5′-ITR and 3′-ITR regions compared between clade I, clade IIa, and clade IIb MPXV viruses (3). Notably, these gene losses and gains are followed by unique tandem repeats identified in the present study. Studies have shown that genetic diversity, gene loss, and gain in the ITR and regions following the ITR are generally high in poxviruses (4–6). Also, it is worth noting that most of the genes in this region regulate innate host immunity and determine host range (4–6). However, tandem repeats of different lengths (4-bp, 54-bp, and 70-bp repeats) have been documented at the ends of the vaccinia virus (VACV) genome (7, 8), and a six-nucleotide repeat (5′-ACAGAT-3′ has been documented at the 5′ ends of the B11R gene of VACV Dryvax(DVX)_204 (9). Also, 34-bp/55-bp, 47-bp, 31- to 32-bp, and 17- to 48-bp tandem repeats have been detected in Pigeonpox virus (FeP2), Penguinpox virus (PEPV), Fowlpox virus (FPVUS), and Canarypox virus (CNPV), respectively (10, 11). Similarly, short interspersed elements (SINEs) repeats have been found in taterapox virus (TATV) (12), and long interspersed nuclear element-1 (LINE-1) repeats have been documented in camelpox, cowpox, monkeypox, and vaccinia viruses (13). Finally, the presence of telomere junctions and hairpin structures formed by AT-rich tandem direct repeats in the ITR regions of poxviruses is well known (14). From these, it is clear that the presence of tandem repeats in the genome of poxviruses is a common feature, but it is noteworthy that these specific sequences found in MPXVs (AACTAACTTATGACTT) in this present study are not present in other poxviruses.

In conclusion, different groups of MPXVs contain unique tandem repeats with different copy numbers, and these may be expected to accommodate the genetic diversity of the virus. However, the functional significance of these unique tandem repeats is unknown, so experimental studies on these should be done in the future. Furthermore, the present study also revealed that among the tandem repeats reported in human and rodent genomes, 38 and 32 tandem repeats are in clade IIb (B) MPXV, respectively, and the functional significance of these tandem repeats should also be studied. Also, the difference in the copy numbers of these tandem repeats in noncoding genomic regions (AACTAACTTATGACTT) may affect the expression of early proteins such as chemokine binding protein, J2L tumor necrosis factor receptor (CrmB), and Ankyrin (Cop-C19L) J3L (which present in the downstream of these repeats) that involve in the host immunity; thereby these repeats copy number difference may contribute to virus transmission and disease outcome. Finally, when developing attenuated or modified MPXV vaccine strains, these repeats (AACTAACTTATGACTT) can be exploited to incorporate foreign proteins (adjuvants/other virus proteins/racking fluorescent proteins such as green fluorescent protein) to carry out vaccine production and virus pathogenesis studies.

### Data availability.

All the data generated in this work is available as supplementary information.
