# Supplementary material for: Unique Tandem Repeats in the Inverted Terminal Repeat Regions of Monkeypox Viruses
Source: Microbiol Spectr. 2023 Mar 28;11(2):e03199-22. doi: 10.1128/spectrum.03199-22 (PMC10101126; doi:10.1128/spectrum.03199-22)
Supplement: Supplemental file 1 — Supplemental material. Download spectrum.03199-22-s0001.pdf, PDF file, 7.8 MB [file spectrum.03199-22-s0001.pdf]

Unique differential copies of Tandem repeats in the ITR regions of Monkeypox viruses

Perumal Arumugam Desingu[1*], K. Nagarajan[2], Nagalingam R. Sundaresan[1]

[1] Department of Microbiology and Cell Biology, Indian Institute of Science, Bengaluru, India
[2]Department of Veterinary Pathology, Madras Veterinary College, Vepery, Chennai, 600007, Tamil Nadu Veterinary and Animal Sciences University (TANUVAS)

Supplementary Materials and Methods

Data curation and Sequence alignment

In this study, sequences of MPXVs were retrieved from NCBI and GISAID databases and aligned using MAFFT 7.407_1 multiple alignment program with parameters such as Gap extend penalty of 0.123 and Gap opening penalty of 1.53[1-3].

Phylogenetic analysis

For phylogenetic inference from MPXV complete genome sequences aligned by MAFFT 7.407_1, the aligned sequences were selected using BMGE 1.12_1[4] (Block Mapping and Gathering with Entropy) and the phylogenetic tree was performed in PhyML 3.3_1. The midpoint of the phylogenetic topology is shown in the midpoint-rooted tree. Phylogenetic analysis of complete genome, ITR, and tandem repeats of MPXVs in this study was performed using PhyML 3.3_1 (Galaxy Version 3.3_1)/ngphylogeny.fr[3,5,6] after aligning in MAFFT 7.407_1. GTR (Evolutionary model), discrete gamma model with categories (n=4), Empirical (Equilibrium Frequencies), SPR (Subtree Pruning and Regraphing) with tree topology search with optimizing parameters such as branch length, tree topology, and model parameter, and test the branch support of approximate Bayes branch was utilized to perform phylogenetic analysis in PhyML 3.3_1. After this, these phylogenetic trees were visualized using the interactive tree of life (iTOL) v5[7].

Similarity Plot analysis

In this study, Similarity Plot analysis for 5'-ITR and 3'-ITR regions of MPXVs was performed using SimPlot 3.5.1[8]. The 5'-ITR and 3'-ITR regions of MPXVs were first aligned in MAFFT 7.407_1 before they were exported to SimPlot 3.5.1 for subsequent analysis. Next, SimPlot analysis of MPXVs was carried out using the Kimura (2-parameter) method with 100 base pairs of the window at a 30 base-pair step.

Tandem repeats identification

Tandem repeats in monkeypox virus ON676705.1/MPXV_USA_2022_UT001 detected using rmblast and human and rodent (mice and rat) genome reference on RepeatMasker Web Server (https://www.repeatmasker.org/)

Supplementary Figures and Legends

Supplementary Figure 1. (A-B) Phylogenetic analysis for the 5'-ITR (**A**) and 3'-ITR (**B**) regions of MPXV viruses. Clade I, Clade IIa, Clade IIb (A), and Clade IIb (B) MPXVs formed a distinct group in both the 5'-ITR (**A**) and 3'-ITR (**B**) regions.

Supplementary Figure 2: (**A**) Similarity plots display the ≈600bp long region of nucleotide sequence diversity (gap) in the 5'-ITR regions between Clade I, Clade IIa, Clade IIb (A), and Clade IIb (B) MPXVs. NC_003310.1/MPXV/Zaire-96-I-16 (Clade I) was used as the query reference sequence. (**B**) Phylogenetic analysis for 5'-ITR regions containing tandem repeats. Clade I, Clade IIa, Clade IIb (A), and Clade IIb (B) MPXVs formed a separate group. (Details of the sequences used for this analysis are presented in **Supplementary Data 1 and 2**).

References

1 Lemoine, F. *et al.* NGPhylogeny.fr: new generation phylogenetic services for non-specialists. *Nucleic acids research* **47**, W260-W265, doi:10.1093/nar/gkz303 (2019).

2 Katoh, K. & Standley, D. M. MAFFT multiple sequence alignment software version 7: improvements in performance and usability. *Mol Biol Evol* **30**, 772-780, doi:10.1093/molbev/mst010 (2013).

3 Fabien Mareuil, O. D.-A., Hervé Ménager. doi:https://doi.org/10.7490/f1000research.1114334.1) (2017).

4 Criscuolo, A. & Gribaldo, S. BMGE (Block Mapping and Gathering with Entropy): a new software for selection of phylogenetic informative regions from multiple sequence alignments. *BMC Evol Biol* **10**, 210, doi:10.1186/1471-2148-10-210 (2010).

5 Guindon, S. *et al.* New algorithms and methods to estimate maximum-likelihood phylogenies: assessing the performance of PhyML 3.0. *Syst Biol* **59**, 307-321, doi:10.1093/sysbio/syq010 (2010).

6 Lemoine, F. *et al.* Renewing Felsenstein's phylogenetic bootstrap in the era of big data. *Nature* **556**, 452-456, doi:10.1038/s41586-018-0043-0 (2018).

7 Letunic, I. & Bork, P. Interactive Tree Of Life (iTOL) v5: an online tool for phylogenetic tree display and annotation. *Nucleic Acids Res* **49**, W293-W296, doi:10.1093/nar/gkab301 (2021).

8 Desingu, P. A., Nagarajan, K. & Dhama, K. SARS-CoV-2 gained a novel spike protein S1-N-Terminal Domain (S1-NTD). *Environmental research* **211**, 113047, doi:10.1016/j.envres.2022.113047 (2022).

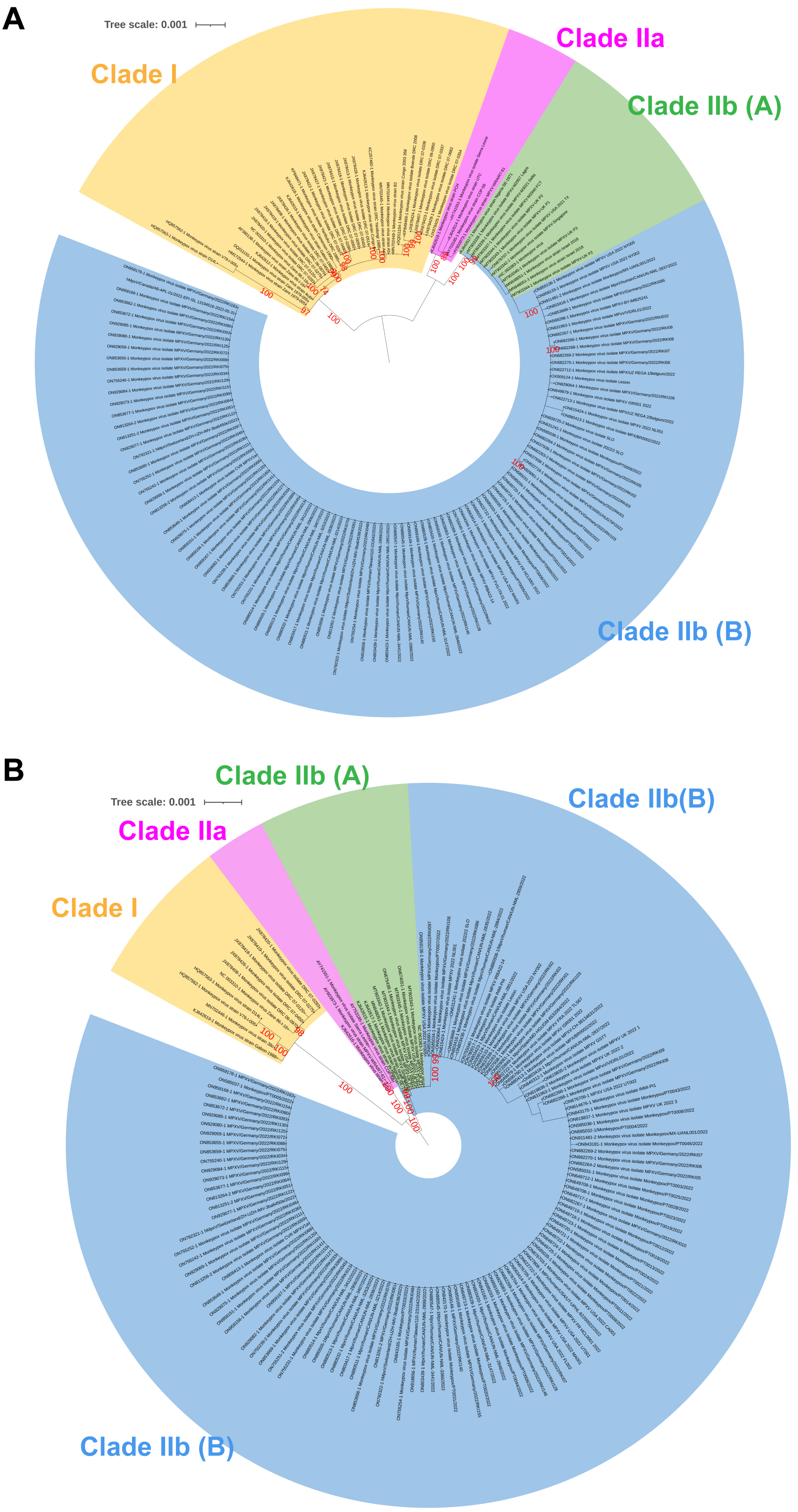

Supplementary Figure 1

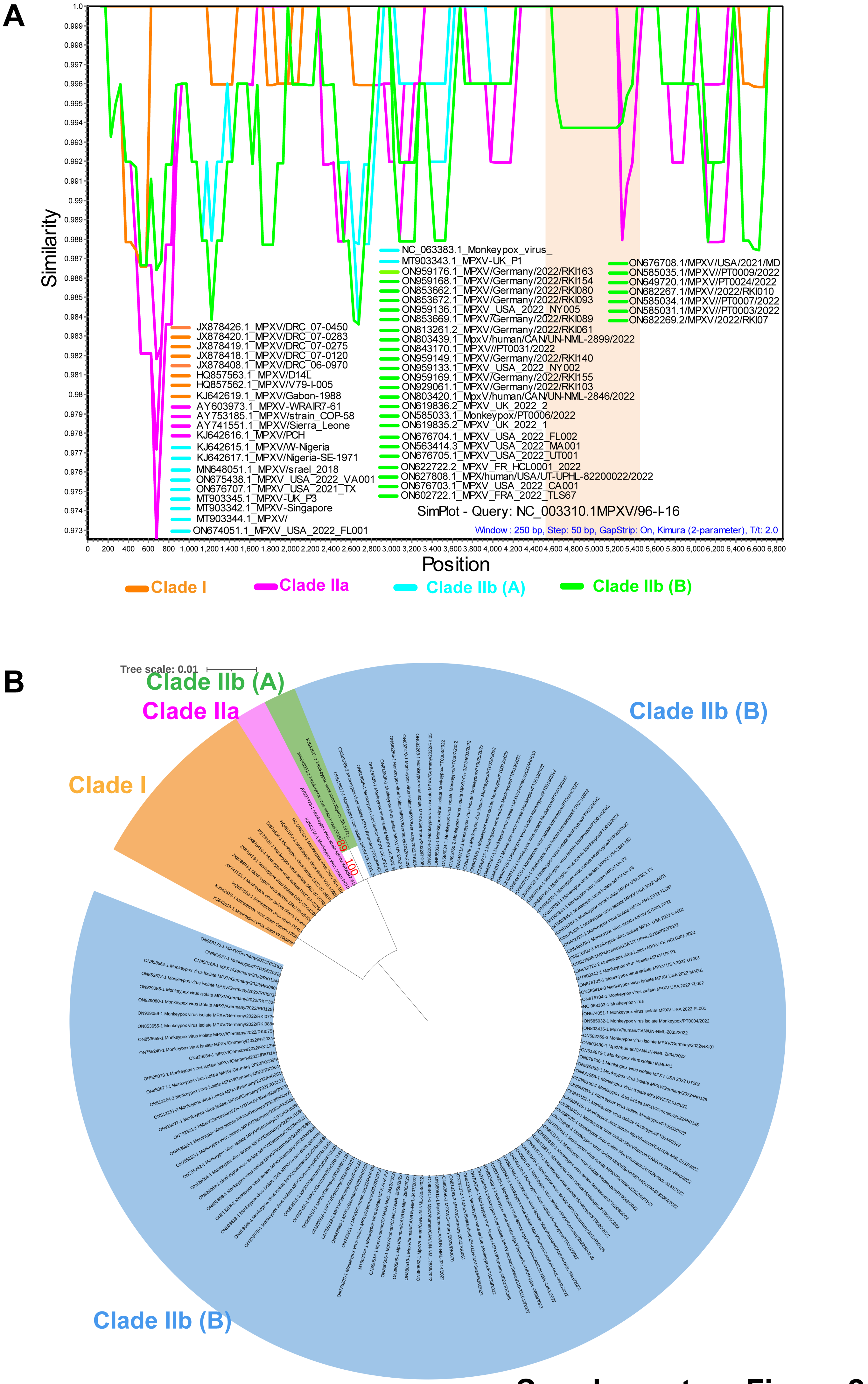

Supplementary Figure 2

Supplementary Table 1: Tandem repeats similar to those present in the human genome present in MPXVs-2022 (ON676705.1/MPXV_USA_2022_UT001) viruses are tabulated here.

S.No.	pValue	Score	Method	Begin	End	Repeat	Type	Begin	End
1.	-	13	RMasker/TRF	1818	1853	+ (TAGTAT)n	Simple_repeat	1	33
2.	-	14	RMasker/TRF	6430	6510	+ (TCATTA)n	Simple_repeat	1	79
3.	-	13	RMasker/TRF	6439	6471	+ (TTA)n	Simple_repeat	1	33
4.	-	11	RMasker/TRF	7482	7543	+ (TAATAAT)n	Simple_repeat	1	64
5.	-	18	RMasker/TRF	10421	10451	+ (ATTAG)n	Simple_repeat	1	32
6.	-	12	RMasker/TRF	11343	11372	+ (TCAA)n	Simple_repeat	1	29
7.	-	11	RMasker/TRF	12033	12067	+ A-rich	Low_complexity	1	35
8.	1.40e-22	254	WUBlastX	29281	29664	- Gypsy-4_PPM_1p	LTR/Gypsy	82	210
9.	-	13	RMasker/TRF	29747	29774	+ (CCATT)n	Simple_repeat	1	29
10.	1.40e-11	22	WUBlastX	29774	31120	- UN-48139970_Ame	DNA/hAT-Ac	575	1038
11.	8.00e-06	53	WUBlastX	29786	30385	- UN-48139970_Ame	DNA/hAT-Ac	868	1081
12.	-	11	RMasker/TRF	33679	33720	+ (AATTAT)n	Simple_repeat	1	39
13.	-	11	RMasker/TRF	41109	41156	+ (TTTCAAT)n	Simple_repeat	1	44
14.	-	14	RMasker/TRF	48111	48173	+ (ATAGT)n	Simple_repeat	1	62
15.	-	13	RMasker/TRF	58605	58656	+ (AAT)n	Simple_repeat	1	51
16.	-	16	RMasker/TRF	116650	116690	+ (TTATTA)n	Simple_repeat	1	41
17.	-	15	RMasker/TRF	125933	125977	+ A-rich	Low_complexity	1	47
18.	-	20	RMasker/TRF	133079	133100	+ (T)n	Simple_repeat	1	22
19.	-	49	RMasker/TRF	136501	136557	+ (ATC)n	Simple_repeat	1	57
20.	-	13	RMasker/TRF	139613	139643	+ (TATTTG)n	Simple_repeat	1	33
21.	-	43	RMasker/TRF	140093	140132	+ (ATAACAATT)n	Simple_repeat	1	40
22.	-	13	RMasker/TRF	140136	140174	+ (TTATAA)n	Simple_repeat	1	38
23.	9.30e-05	64	WUBlastX	140373	140918	- Polinton-1_NV_a	DNA/Maverick	28	205
24.	3.20e-03	69	WUBlastX	140532	140960	- Polinton-1_DEu	DNA/Maverick	20	152
25.	-	14	RMasker/TRF	141964	142006	+ (AATGAT)n	Simple_repeat	1	43
26.	-	44	RMasker/TRF	146832	146903	+ (ATTTTAT)n	Simple_repeat	1	78
27.	-	67	RMasker/TRF	150532	150599	+ (TATGATGGA)n	Simple_repeat	1	69
28.	3.20e-03	21	WUBlastX	157185	157280	+ UN-48139970_Ame	DNA/hAT-Ac	567	598
29.	-	20	RMasker/TRF	160525	160565	+ A-rich	Low_complexity	1	41
30.	-	26	RMasker/TRF	163161	163185	+ (TAAC)n	Simple_repeat	1	25
31.	-	12	RMasker/TRF	166061	166116	+ A-rich	Low_complexity	1	57
32.	-	59	RMasker/TRF	169693	169745	+ (CAGATA)n	Simple_repeat	1	53
33.	-	54	RMasker/TRF	173247	173292	+ (AT)n	Simple_repeat	1	46
34.	-	31	RMasker/TRF	174494	174522	+ (GATGAA)n	Simple_repeat	1	29
35.	4.70e-01	78	WUBlastX	178151	178348	+ UN-48139970_Ame	DNA/hAT-Ac	572	636
36.	-	172	RMasker/TRF	179052	179200	+ (ATATACATT)n	Simple_repeat	1	149
37.	-	20	RMasker/TRF	186842	186900	+ A-rich	Low_complexity	1	57
38.	-	13	RMasker/TRF	195312	195347	+ (CTAATA)n	Simple_repeat	1	33

Supplementary Table 2: Tandem repeats similar to those present in the Rodent (Rat and Mice) genome present in MPXVs-2022 (ON676705.1/MPXV_USA_2022_UT001) viruses are tabulated here.

S.No.	Score	Method	Begin	End	Repeat	Type	Begin	End
1	13	RMasker/TRF	1818	1853	+(TAGTAT)n	Simple_repeat	1	33
2	14	RMasker/TRF	6430	6438	+(TCATTA)n	Simple_repeat	1	13
3	13	RMasker/TRF	6439	6471	+(TTA)n	Simple_repeat	1	33
4	14	RMasker/TRF	6472	6510	(TCATTA)n	Simple_repeat	1	79
5	11	RMasker/TRF	7482	7543	(TAATAAT)n	Simple_repeat	1	64
6	18	RMasker/TRF	10421	10451	(ATTAG)n	Simple_repeat	1	32
7	12	RMasker/TRF	11343	11372	(TCAA)n	Simple_repeat	1	29
8	11	RMasker/TRF	12033	12067	A-rich	Low_complexity	1	35
9	13	RMasker/TRF	29747	29774	(CCATT)n	Simple_repeat	1	29
10	11	RMasker/TRF	33679	33720	(AATTAT)n	Simple_repeat	1	39
11	11	RMasker/TRF	41109	41156	(TTTCAAT)n	Simple_repeat	1	44
12	14	RMasker/TRF	48111	48173	(ATAGT)n	Simple_repeat	1	64
13	13	RMasker/TRF	58605	58656	(AAT)n	Simple_repeat	1	51
14	16	RMasker/TRF	116650	116690	(TTATTA)n	Simple_repeat	1	41
15	15	RMasker/TRF	125933	125977	A-rich	Low_complexity	1	47
16	20	RMasker/TRF	133079	133100	(T)n	Simple_repeat	1	22
17	49	RMasker/TRF	136501	136557	(ATC)n	Simple_repeat	1	57
18	13	RMasker/TRF	139613	139643	(TATTTG)n	Simple_repeat	1	33
19	43	RMasker/TRF	140093	140132	(ATAACAATT)n	Simple_repeat	1	40
20	13	RMasker/TRF	140136	140174	(TTATAA)n	Simple_repeat	1	38
21	14	RMasker/TRF	141964	142006	(AATGAT)n	Simple_repeat	1	43
22	44	RMasker/TRF	146832	146903	(ATTTTAT)n	Simple_repeat	1	78
23	67	RMasker/TRF	150532	150599	(TATGATGGA)n	Simple_repeat	1	69
24	20	RMasker/TRF	160525	160565	A-rich	Low_complexity	1	41
25	26	RMasker/TRF	163161	163185	(TAAC)n	Simple_repeat	1	25
26	12	RMasker/TRF	166061	166116	A-rich	Low_complexity	1	57
27	59	RMasker/TRF	169693	169745	(CAGATA)n	Simple_repeat	1	53
28	54	RMasker/TRF	173247	173292	(AT)n	Simple_repeat	1	46
29	31	RMasker/TRF	174494	174522	(GATGAA)n	Simple_repeat	1	29
30	172	RMasker/TRF	179052	179200	(ATATACATT)n	Simple_repeat	1	149
31	20	RMasker/TRF	186842	186900	A-rich	Low_complexity	1	57
32	13	RMasker/TRF	195312	195347	(CTAATA)n	Simple_repeat	1	33
